# Maternal opioids age-dependently impair neonatal respiratory control networks

**DOI:** 10.3389/fphys.2023.1109754

**Published:** 2023-03-16

**Authors:** Sarah A. Beyeler, Robyn Naidoo, Nina R. Morrison, Emilee A. McDonald, David Albarrán, Adrianne G. Huxtable

**Affiliations:** ^1^ Department of Biology, Institute of Neuroscience, University of Oregon, Eugene, OR, United States; ^2^ Department of Human Physiology, University of Oregon, Eugene, OR, United States

**Keywords:** maternal opioid use, neonatal respiratory control, *in vitro* electrophysiology, perinatal development, neonatal abstinence syndrome

## Abstract

Infants exposed to opioids *in utero* are an increasing clinical population and these infants are often diagnosed with Neonatal Abstinence Syndrome (NAS). Infants with NAS have diverse negative health consequences, including respiratory distress. However, many factors contribute to NAS, confounding the ability to understand how maternal opioids directly impact the neonatal respiratory system. Breathing is controlled centrally by respiratory networks in the brainstem and spinal cord, but the impact of maternal opioids on developing perinatal respiratory networks has not been studied. Using progressively more isolated respiratory network circuitry, we tested the hypothesis that maternal opioids directly impair neonatal central respiratory control networks. Fictive respiratory-related motor activity from isolated central respiratory networks was age-dependently impaired in neonates after maternal opioids within more complete respiratory networks (brainstem and spinal cords), but unaffected in more isolated networks (medullary slices containing the preBötzinger Complex). These deficits were due, in part, to lingering opioids within neonatal respiratory control networks immediately after birth and involved lasting impairments to respiratory pattern. Since opioids are routinely given to infants with NAS to curb withdrawal symptoms and our previous work demonstrated acute blunting of opioid-induced respiratory depression in neonatal breathing, we further tested the responses of isolated networks to exogenous opioids. Isolated respiratory control networks also demonstrated age-dependent blunted responses to exogenous opioids that correlated with changes in opioid receptor expression within a primary respiratory rhythm generating region, the preBötzinger Complex. Thus, maternal opioids age-dependently impair neonatal central respiratory control and responses to exogenous opioids, suggesting central respiratory impairments contribute to neonatal breathing destabilization after maternal opioids and likely contribute to respiratory distress in infants with NAS. These studies represent a significant advancement of our understanding of the complex effects of maternal opioids, even late in gestation, contributing to neonatal breathing deficits, necessary first steps in developing novel therapeutics to support breathing in infants with NAS.

## 1 Introduction

Maternal opioid use is increasing, thereby increasing the number of infants born exposed to opioids *in utero* >6 fold (Batra et al., 2021). Approximately 80 infants are born daily exposed to *in utero* opioids (Centers for Disease Control and Prevention, 2022), with an infant being born approximately every 15 min exposed to maternal opioids (National Institute on Drug Abuse, 2019). Yet, these infants remain an understudied population in the opioid crisis (Kocherlakota, 2014). Maternal opioids cross the placenta (Nanovskaya et al., 2008), directly disrupting *in utero* fetal development (including neural tube formation; Williams et al., 1991), with the most severe effects occurring late in gestation when opioids accumulate in the fetus (Conradt et al., 2018). Infants born after *in utero* opioid exposure are often diagnosed with Neonatal Abstinence Syndrome (NAS), which presents as a diverse set of symptoms, including sleep disturbances, temperature instability, tremors, seizures, high pitched crying, excessive yawning, tachypnea (McQueen and Murphy-Oikonen, 2016), apneas, and respiratory deficits (Fältmarch et al., 2019). Although these symptoms are thought to result from dysfunctions in the central and autonomic nervous systems, and gastrointestinal system, their etiology remains poorly understood (McQueen and Murphy-Oikonen, 2016). Infants with NAS often require exogenous opioids to manage withdrawal symptoms (Mangat et al., 2019), though these additional opioids may have further disruptive effects on infant health. Respiratory deficits in these infants are a significant clinical problem and require additional clinical interventions for infant survival (Fältmarch et al., 2019). However, the etiology of these respiratory deficits in infants with NAS remains unclear, restricting the ability to develop treatments to facilitate infant breathing.

The direct effect of maternal opioids on neonatal breathing are difficult to study in humans, as maternal polysubstance use, poor nutrition, and stress often accompany maternal opioid use and may contribute to symptomology in infants with NAS, including respiratory deficits (reviewed in Farid et al., 2008; Conradt et al., 2018). The rodent represents an ideal model to directly study the impact of maternal opioids since it allows for control of these confounding factors and facilitates the use of more invasive techniques to identify the central origins of breathing deficits. In a rat model of maternal opioids starting at conception, maternal opioids induced reorganization of neonatal central respiratory networks (Gourévitch et al., 2016), yet the impact on neonatal breathing behaviors was not assessed. We developed a novel, late gestation opioid model in rodents to test the impact of maternal opioids on respiratory network maturation without disrupting early developmental processes (such as neurogenesis, gliogenesis, myelination, and synaptogenesis; reviewed in Farid et al., 2008; Hauser and Knapp, 2018). In this late gestation model, opioid exposure begins at the onset of respiratory rhythm *in utero*, after other critical components of the central nervous system have developed (Hocker et al., 2021). Using this model, maternal opioids acutely destabilized neonatal breathing (increased apneas, increased breathing variability, and blunted the hypoxic ventilatory decline) and blunted opioid-induced respiratory depression immediately after birth, before breathing normalized with age (Hocker et al., 2021). Given the similarities in breathing deficits in neonates after late gestational maternal opioids (Hocker et al., 2021) and human infants with NAS (Fältmarch et al., 2019), maternal opioids may disrupt perinatal maturation of the central respiratory system and contribute to breathing deficits. Yet, where within the heterogeneous and anatomically distributed respiratory system maternal opioids directly influence perinatal maturation remains unknown. To identify the central origins of breathing deficits in neonates after maternal opioids, we first assessed isolated neonatal central respiratory networks known to control breathing, independent of peripheral influences. We tested the hypothesis that maternal opioids impair neonatal central respiratory networks. Methadone was the opioid of choice since it is commonly prescribed to mothers with opioid use disorder (Kocherlakota, 2014; Davis et al., 2018), in part due to its long half-life (Kraft et al., 2016). Others have demonstrated impaired ontogenesis in the rat with this same dose and type of opioid (reviewed in Farid et al., 2008) and it mimics aspects of the high/rush associated with opioid use (Hauser and Knapp, 2018). Further, infants with NAS show improved neonatal outcomes with methadone treatment (Patrick et al., 2014; Young et al., 2015; Davis et al., 2018). Here, we are extending our previous work (Hocker et al., 2021) to triangulate on the central deficits contributing to impaired neonatal breathing in this model of late gestation opioids. Collectively, this study demonstrates maternal opioids age-dependently impair isolated neonatal central respiratory networks, suggesting central impairments may contribute to neonatal breathing deficits after maternal opioids. A component of the central mechanism contributing to these impairments involves opioid receptors. Opioid receptor antagonists suggest lingering opioids in neonatal central respiratory networks contribute to reduced fictive respiratory activity. Yet, isolated central respiratory networks from neonates after maternal opioids had blunted responses to acute opioids, similar to breathing responses shown previously (Hocker et al., 2021). This blunted response to acute opioids may be due to reduced expression of opioid receptors in a key respiratory rhythm generating region in the neonatal medulla, the preBötzinger complex (preBötC), after maternal opioids. However, central respiratory network impairments extend beyond the preBötC, since further isolated neonatal central respiratory networks showed no impairments. Emergence of a distinct respiratory pattern in neonates after maternal opioids further supports impairments to regions beyond the preBötC and highlights the need to further investigate regions associated with respiratory pattern. In summary, maternal opioids impair neonatal central respiratory networks and these central impairments likely contribute to neonatal breathing deficits after maternal opioids (Hocker et al., 2021). This study advances our understanding of the etiology of respiratory deficits in infants after maternal opioids and is a needed step toward developing novel treatments to support breathing in infants with NAS.

## 2 Materials and methods

All experiments were approved by the Institutional Animal Care and Use Committee at the University of Oregon and conformed to the policies of the National Institutes of Health, Guide for the Care and Use of Laboratory Animals. Timed pregnant Sprague-Dawley dams (E17) were purchased in pairs from commercial vendors (Envigo, colony 231 and 202; Charles River, stock H41) and monitored daily until giving birth. Rats were housed under standard conditions (12:12 h light/dark cycle) with food and water *ad libitum*.

### 2.1 Maternal opioid exposure

Maternal opioid exposure was administered as described previously (Hocker et al., 2021). This opioid exposure model facilitates investigating the effects of maternal opioids on maturation of the respiratory control system, without impacting the known effects of maternal opioids on critical neurodevelopmental processes, such as cell genesis, myelination and synaptogenesis (reviewed in Boggess and Risher, 2022). In brief, maternal opioid exposure (methadone hydrochloride in sterile saline, 5 mg/kg, subcutaneous, Sigma-Aldrich, cat#1095905) began at embryonic day 17 (E17), the onset of respiratory rhythm generation *in utero* (Greer et al., 1992; Pagliardini et al., 2003). From E17 onward, dams were injected with methadone daily and maternal health monitored for at least 1 h post-injection. Maternal opioid exposure continued until postnatal day 5 (P5). To control for maternal care, litters were balanced and culled to 12 or fewer pups per dam. Since no differences were seen in neonatal breathing in neonates after maternal no treatment or maternal saline injections (Hocker et al., 2021), neonates after maternal no treatment were used as controls.

### 2.2 Brainstem-spinal cord (BSSC) preparations

To study central deficits in isolated respiratory networks from neonates (postnatal day 0–5, P0-5) after maternal opioids, we isolated neonatal brainstems and spinal cords, which contain essential components of central respiratory networks, and recorded spontaneous respiratory-related motor activity (Greer et al., 1991; reviewed in Johnson et al., 2012). Fictive respiratory activity from isolated brainstem-spinal cords (BSSCs) was assessed in neonates after maternal no treatment (P0-1 = 3 male, 2 female, P2 = 2 male, 3 female, P3-5 = 3 male, 2 female) and neonates after maternal opioids (P0-1 = 3 male, 3 female, P2 = 3 male, 3 female, P3-5 = 2 male, 3 female). The responses to opioid receptor antagonism and agonism were measured in a separate set of experiments. Respiratory activity was assessed in isolated BSSCs after mu-opioid receptor antagonism (naloxone 10 µM, 2 h, Sigma-Aldrich, cat#N7758) in neonates after maternal no treatment (P0-1 = 3 male, 3 female, P2 = 3 male, 3 female, P3-5 = 3 male, 3 female) and neonates after maternal opioids (P0-1 = 3 male, 3 female, P2 = 2 male, 3 female, P3-5 = 3 male, 3 female). Respiratory activity was also assessed in response to acute, exogenous opioids in isolated BSSCs after mu-opioid receptor agonism (methadone 10 µM, 2 h, Sigma-Aldrich, cat#1095905) in neonates after maternal no treatment (P0-1 = 3 male, 3 female, P2 = 2 male, 3 female, P3-5 = 3 male, 3 female) and neonates after maternal opioids (P0-1 = 3 male, 3 female, P2 = 3 male, 3 female, P3-5 = 2 male, 2 female).

Brainstem-spinal cord (BSSC) preparations were prepared from neonates (P0-5), as described previously (Suzue, 1984; Greer et al., 1992; Morrison et al., 2019). In brief, neonates were anesthetized with isoflurane and decerebrated. The thoracic and cervical spinal cord regions were isolated and placed in artificial cerebrospinal fluid (aCSF), containing the following (in mM): 120 NaCl, 3 KCl, 1.25 NaH_2_PO_4_, 1.0 CaCl_2_, 2.0 MgSO_4_, 26 NaHCO_3_, 20 D-glucose, equilibrated with 95% O_2_/5% CO_2_. A dorsal laminectomy revealed the spinal cord before removal of the lungs and heart. A ventral laminectomy isolated BSSCs and brainstems were transected at the pontomedullary junction. Isolated BSSCs were pinned ventral side up in a recording chamber (8.2 ml volume, 28°C) with aCSF (continuously bubbled with 95% O_2_/5% CO_2_) and circulated *via* a peristaltic pump (8–10 ml/min, MINIPULS3, Gilson, Inc., Middleton, WI). Respiratory activity was recorded from the 4th or 5th cervical nerve rootlets using glass suction electrodes (internal diameter 70–80 μm). Recordings were amplified (x1000–10 k), bandpass-filtered (0.1 Hz to 1 kHz; Model 1700 Differential AC amplifier, A-M Systems, Carlsburg, WA), integrated (*τ* = 50 m) and rectified (LabChart, Version 8.1, ADInstruments). Preparations equilibrated (40–50 min) prior to recording baseline activity. Neurograms illustrate respiratory activity from baseline through 120 min, whereby changes over time are compared within treatment at baseline (30-min averages, baseline = 0–30 min) and 120 min (30-min averages = 90–120 min), as described in Olsson et al. (2003); Tsuzawa et al. (2015); Kotani et al. (2004). Data are presented as the percent change from within treatment baseline (% change from baseline; $\left.\frac{120\min average-baseline\;average}{baseline\;average}*100\right)$ , as described in Gumnit et al. (2022); Cook-Snyder et al. (2019). Throughout the manuscript, asterisk (*) symbols denote significant differences from baseline within a treatment group, pound (#) signs highlight significant differences between treatment groups, and ampersand (&) symbols represent significant differences between ages. For experiments with the mu-opioid receptor antagonist (naloxone, 10 µM) or opioid receptor agonist (methadone, 10 µM), BSSCs equilibrated (40–50 min) and antagonists or agonists were bath-applied following baseline (0–30 min = baseline). Responses to antagonists and agonists were monitored for 120 min, whereby changes from baseline were analyzed at 120 min (90–120 min = 120 min average). The percent change from baseline was calculated as described above.

### 2.3 Immunohistochemistry

Given the changes in respiratory activity after acute, exogenous opioids, we next sought to view neonatal opioid receptor expression in a key respiratory rhythm generating region, the preBötC (Smith et al., 1991), after maternal opioids. Opioid receptor expression in the neonatal preBötC were characterized at three postnatal ages: at a critical period immediately post-birth when neonatal breathing is acutely destabilized and opioid-induced respiratory depression is blunted after maternal opioids (P0), during the first week of life when breathing normalized after maternal opioids and neonates continued receiving opioids through breast milk (P4), and during the second week of life after maternal opioid exposure ceased (P11). Before determining the effect of maternal opioids on opioid receptor expression, mu-opioid receptors in the neonatal preBötzinger Complex (preBötC) during typical postnatal development were first characterized using immunohistochemistry in neonates (maternal no treatment neonates: P0 = 3 male, 3 female; P4 = 3 male, 3 female; P11 = 3 male, 3 female). In a separate set of experiments, the impact of maternal opioids on expression of mu-opioid receptors in the preBötC was determined with immunohistochemistry at the same three ages in neonates after maternal no treatment (P0 = 3 male, 3 female; P4 = 3 male, 3 female; P11 = 3 male, 3 female) and neonates after maternal opioids (P0 = 3 male, 3 female; P4 = 3 male, 3 female; P11 = 3 male, 3 female).

Immunohistochemistry experiments were performed similar to our previous work (Hocker et al., 2019), but were optimized for neonatal medullary tissue. In brief, neonates (P0, P4 and P11) after maternal no treatment or maternal opioids were perfused (transcardiac) with cold phosphate-buffered saline (PBS, pH 7.4) and 4% paraformaldehyde (pH 7.4 in PBS). Brain tissue was extracted and immersed in paraformaldehyde for 24hrs before being transferred to PBS until vibratome sectioning (Leica VT 1200S vibratome). The medullary slices containing the preBötC were selected from -0.35 mm to -0.45 mm from the rostral edge of the inferior olive (Ruangkittisakul et al., 2006). We further identified medullary slices containing the preBötC using 10x images with the semi-compact nucleus ambiguous and bright expression of NK1R in the ventral lateral region, as described previously (Gray et al., 1999). Transverse medullary sections (40 µm) were washed in PBS, blocked with PBS, 0.3% Triton, and 1% BSA (2 h, room temperature) to prevent non-specific antibody binding. Medullary slices were incubated in a buffer solution (PBS with 0.3% triton, 0.01% BSA at room temperature for 16 h) with the following primary antibodies: 1) Guinea pig anti-substance p receptor (1:1000, Millipore AB15810) to identify preBötzinger Complex (preBötC) neurons and 2) Rabbit anti-mu-opioid receptor (1:250, Millipore, AB1774) to label mu-opioid receptors. After primary antibody incubation, medullary sections were rinsed (3 times with PBS) and incubated in a buffer solution (PBS with 0.3% Triton, and 0.01% BSA at room temperature for 2hrs) with the following secondary antibodies: 1) Goat anti-guinea pig 647 IgG (1:1000, Invitrogen, A21450; used when studying typical postnatal expression of mu-opioid receptors) or Goat anti-guinea pig 488 IgG (1:1000, Invitrogen, A11073; used to study changes in mu-opioid receptors after maternal opioids) to label NK1R primary antibodies, and 2) Donkey anti-rabbit 555 IgG (1:1000, Invitrogen, A31572) to label mu-opioid receptor primary antibodies. Binding of secondary antibodies to non-specific epitopes was assessed in medullary sections as described above, but with the omission of primary antibodies. Background tissue fluorescence was assessed in medullary sections as described above, but with the omission of secondary antibodies. Cross reactivity of Guinea pig anti-substance p receptor primary antibody with Donkey anti-rabbit 555 IgG secondary was determined in medullary sections incubated as described above, but with only the Guinea pig anti-substance p receptor primary antibody (no Rabbit anti-mu-opioid receptor primary antibodies) and all relevant secondaries (either Goat anti-guinea pig 647 IgG or Goat anti-guinea pig 488 IgG, and Donkey anti-rabbit 555 IgG). Cross reactivity of the Rabbit anti-mu-opioid receptor primary antibody with Goat anti-guinea pig 647 IgG or Goat anti-guinea pig 488 IgG secondary antibodies was identified in medullary sections incubated as described above, but with only the Rabbit anti-mu-opioid receptor primary antibody (no Guinea pig anti-substance p receptor primary antibodies) and all relevant secondaries (either Goat anti-guinea pig 647 IgG or Goat anti-guinea pig 488 IgG, and Donkey anti-rabbit 555 IgG). Medullary sections were washed and mounted onto charged microscope slides, air-dried, and covered with Prolong Gold (Life Technologies, cat#P10144) to preserve fluorescence. A glass coverslip was placed over the sections, sealed with clear nail polish and stored at 4°C until imaged.

All immunofluorescent images (1024 × 1024 pixels) were acquired using a Zeiss LSM 880 confocal microscope (40x, z-stacks, 0.5 μm increments). To determine the typical expression of mu-opioid receptors in the preBötC during postnatal development, medullary sections from neonates after maternal no treatment (P0, P4, and P11) were all stained and imaged concurrently under identical microscope settings. To assess the effect of maternal opioids on mu-opioid receptor expression in the preBötC, medullary sections from neonates after maternal opioids (P0, P4, and P11) and age-matched neonates after maternal no treatment were stained and imaged concurrently under identical microscope settings. All images were pseudo-colored to visualize NK1 receptors in green and mu-opioid receptors in red. All images for sections run concurrently were taken using identical laser and gain settings, identically adjusted for contrast/brightness, and collapsed into maximum intensity projections using ImageJ open-source software to allow for comparisons between maternal treatments and all ages.

### 2.4 Rhythmically active medullary slice preparations

To further triangulate on where maternal opioids may impair neonatal respiratory networks, we evaluated respiratory activity in rhythmic slice preparations, which further isolate the preBötC. Activity was compared between neonates after maternal no treatment (P0-1 = 3 male, 3 female, P2 = 3 male, 3 female, P3-5 = 3 male, 3 female) and neonates after maternal opioids (P0-1 = 3 male, 3 female, P2 = 3 male, 3 female, P3-5 = 3 male, 3 female). Rhythmically active medullary slices containing the preBötC, hypoglossal motor nucleus, and hypoglossal nerve roots were isolated from P0-5 neonatal rats, as previously described (Ruangkittisakul et al., 2006; Morrison et al., 2019). In brief, isolated BSSCs (as described above) were pinned to a wax chuck and thin slices (200 μm) were cut using a vibratome (Leica VT 1200S vibratome) to visualize anatomical landmarks. Medullary slices were compared to the neonatal rat brainstem atlas and a single 700 μm slice was taken at −0.35 to −0.45 mm from the rostral edge of the inferior olive (Ruangkittisakul et al., 2006). Medullary slices were transferred to a recording chamber (8.2 ml volume, 28°C) with recirculated (10 ml/min) aCSF (continuously bubbled with 95% O_2_/5% CO_2_) *via* a peristaltic pump (MINIPULS3, Gilson, Inc., Middleton, WI). Extracellular potassium was elevated from 3 mM to 9 mM prior to the start of data collection (30–60 min) to offset a loss of tonic excitatory inputs and prevent the gradual slowing of respiratory activity in medullary slice preparations (Smith et al., 1991; Ruangkittisakul et al., 2006; Ballanyi et al., 2009). Respiratory activity was recorded from hypoglossal nerve rootlets using glass suction electrodes (internal diameter 70–80 μm). Recordings were amplified (x10 k), bandpass-filtered (300 Hz to 1 kHz; Model 1700 Differential AC amplifier, A-M Systems, Carlsburg, WA), integrated (*τ* = 50 m) and rectified (LabChart v8.1, ADInstruments). Respiratory activity was assessed as described above in Section 2.2 (Brainstem-spinal cord preparations).

### 2.5 Burst-to-burst variation analysis

To characterize the effect of maternal opioids on respiratory pattern, Poincaré plots were generated in R from periods obtained from Peak Analyses in LabChart, as described previously (Morrison et al., 2019). Burst-to-burst variation in respiratory activity was revealed by graphing the period between two bursts (T_n_) versus the subsequent period (T_n+1_). To quantify the differences between maternal treatments, ratios of the width of the variation perpendicular (SD1) over the line of identity (SD2) were calculated from an ellipse encapsulating 95% of the bursts within a given age and maternal treatment group, as described previously (Brennan et al., 2001; Li and Nattie, 2006; Patrone et al., 2018).

### 2.6 Statistical analyses

**FIGURE 1:**
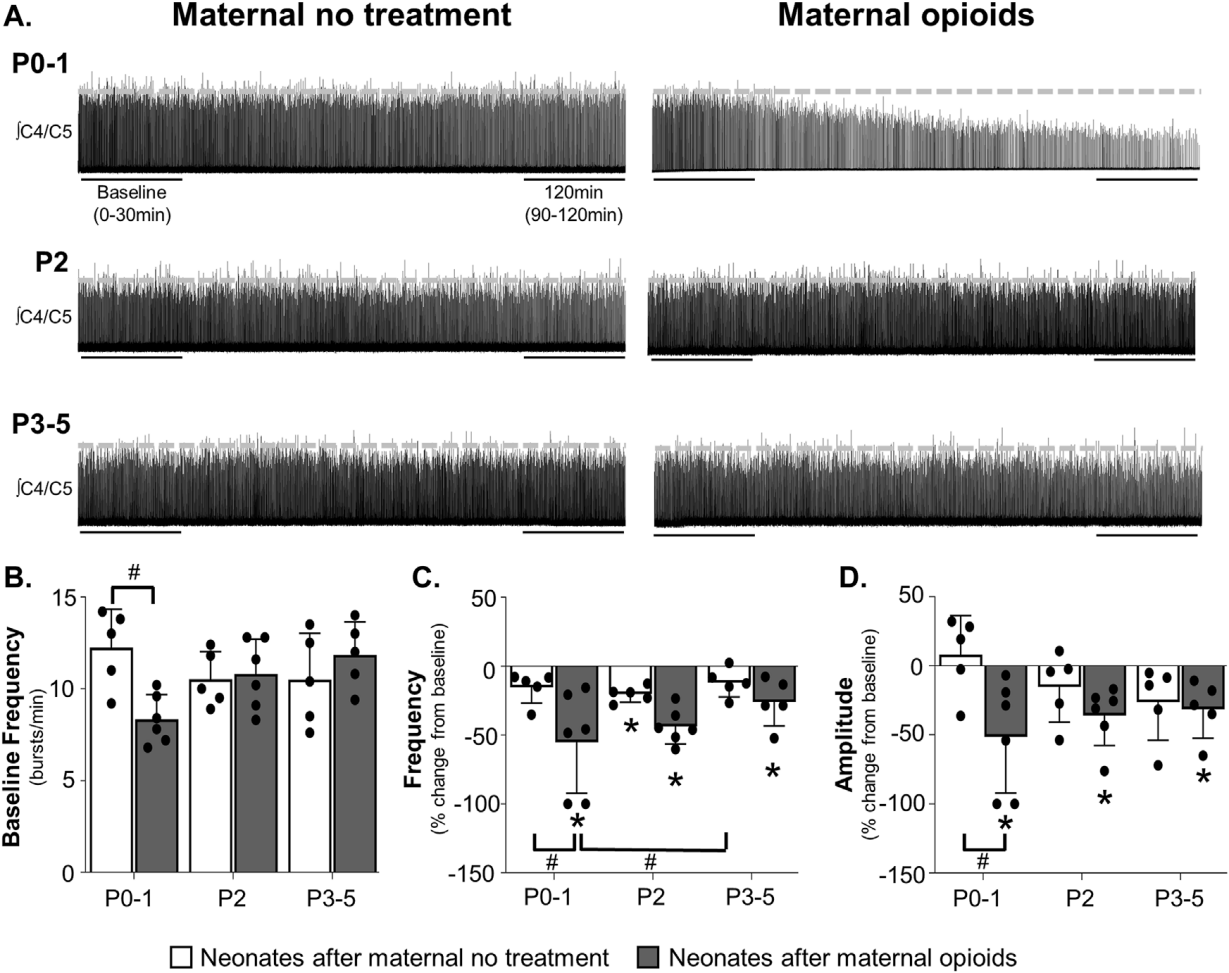
Maternal opioids age-dependently impair neural activity from isolated neonatal respiratory control networks. Representative neurograms **(A)** from isolated respiratory control networks show respiratory activity in neonatal brainstem-spinal cords were maintained in neonates after maternal no treatment at all ages and decreased in P0-1 neonates after maternal opioids. Baseline respiratory burst frequencies [0–30 min, **(B)**] were similar in neonates after maternal no treatment (white bars) at all ages, reduced in P0-1 neonates after maternal opioids (grey bars), and unchanged in older (P2 and P3-5) neonates after maternal opioids. Respiratory burst frequencies **(C)** decreased from baseline in P2 neonates after maternal no treatment and in neonates after maternal opioids at all ages. Respiratory burst frequency decreased in P0-1 neonates after maternal opioids compared to P0-1 and P3-5 neonates after maternal no treatment. Respiratory burst amplitudes **(D)** were maintained in all neonates after maternal no treatment and decreased from baseline in all neonates after maternal opioids. Respiratory burst amplitudes decreased in P0-1 neonates after maternal opioids compared to P0-1 neonates after maternal no treatment (*p < 0.05 different from baseline; ^#^ *p* < 0.05 different from neonates after maternal no treatment; Two- and Three-way ANOVA; Bonferroni post-hoc test.)

**FIGURE 2:**
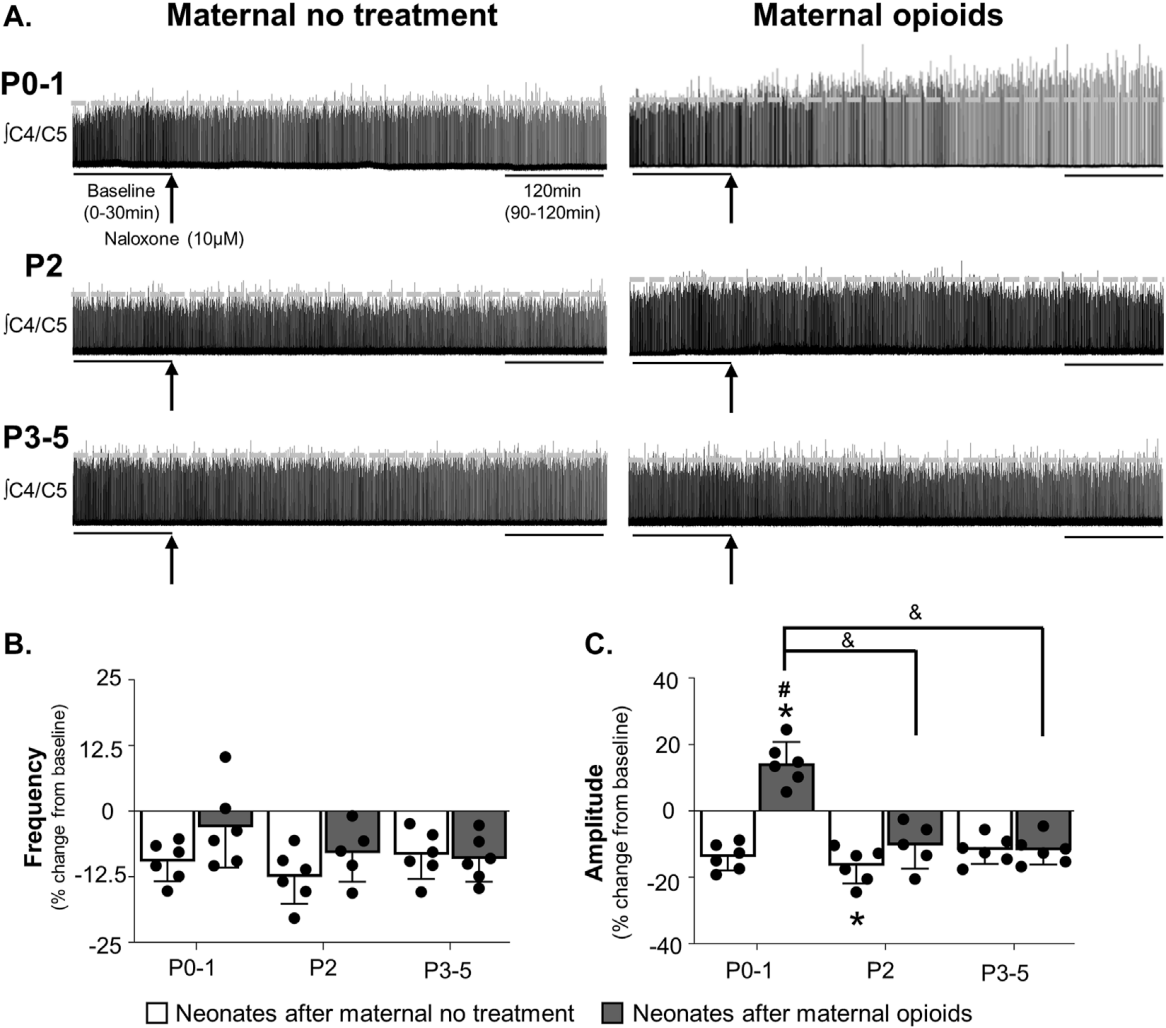
Mu-opioid receptor antagonism increased respiratory burst amplitude, but not frequency, in neonates after maternal opioids. Respiratory neurograms **(A)** show respiratory activity in isolated neonatal brainstem-spinal cords were unchanged after mu-opioid receptor antagonism (arrow, naloxone, 10 µM, bath application) in neonates after maternal no treatment at any age and increased in P0-1 neonates after maternal opioids. Respiratory burst frequencies **(B)** were unchanged from baseline after mu-opioid receptor antagonism in any maternal treatment or age. Respiratory burst amplitudes **(C)** decreased from baseline in P2 neonates after maternal no treatment and increased from baseline in P0-1 neonates after maternal opioids. Respiratory burst amplitude increased in P0-1 neonates after maternal opioids compared to all neonates after maternal no treatment (white bars) and older (P2 and P3-5) neonates after maternal opioids (gray bars) (*p < 0.05 different from baseline; ^#^ *p* < 0.05 different from neonates after maternal no treatment at all ages; ^&^ *p* <0.05 different from neonates after maternal opioids; Three-way ANOVA; Bonferroni post-hoc test).

**FIGURE 3:**
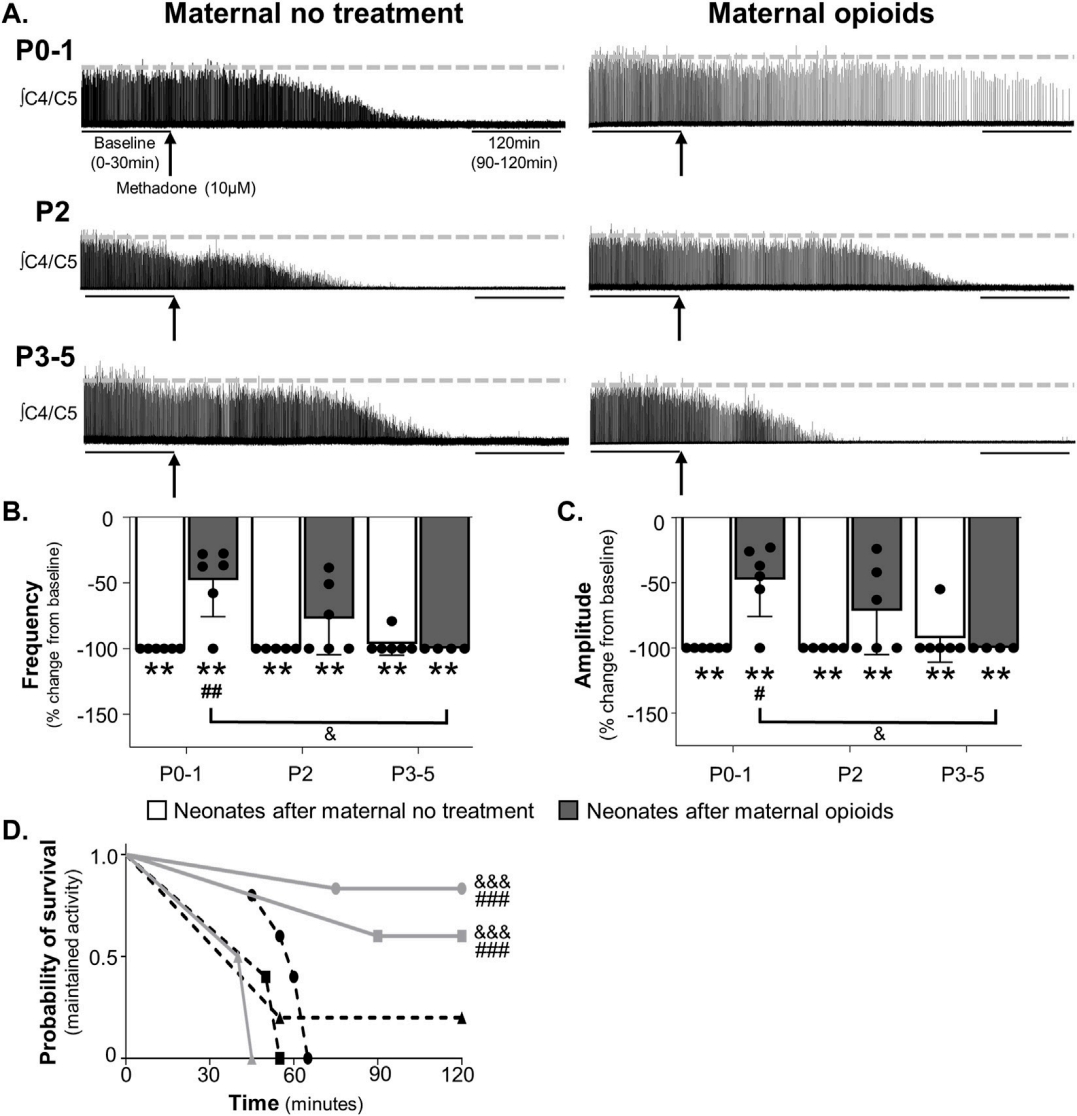
Neonates after maternal opioids age-dependently maintained respiratory activity after acute opioids. Representative neurograms from isolated brainstem-spinal cords **(A)** show respiratory activity was abolished with acute opioids (arrow, methadone, 10 µM, bath application) in neonates after maternal no treatment at all ages, maintained in P0-1 neonates after maternal opioids, and abolished in older neonates (P2 or P3-5) after maternal opioids. Respiratory burst frequencies **(B)** and burst amplitudes **(C)** were abolished in neonates after maternal no treatment at all ages and decreased from baseline in neonates after maternal opioids at all ages. Respiratory burst frequencies and amplitudes were maintained in P0-1 neonates after maternal opioids compared to neonates after maternal no treatment at all ages and older neonates (P3-5) after maternal opioids, but not P2 neonates after maternal opioids. The probability of maintaining respiratory activity after acute opioids **(D)** was greater in P0-1 (circles) and P2 (squares) neonates after maternal opioids (gray lines) than older neonates P3-5 (triangles) after maternal opioids and neonates after maternal no treatment (dotted lines) at all ages (**p <0.001 different from baseline; ^#^ *p* < 0.05, ^##^ *p* < 0.001, ^###^ *p* < 0.0001 different from neonates after maternal no treatment at all ages; ^&^ *p* < 0.05, ^&&&^ *p* < 0.0001 different from neonates after maternal opioids at P3-5; Three-way ANOVA; Bonferroni post-hoc test).

GraphPad Prism software (version 9.3) was used for statistical analyses of *in vitro* electrophysiology experiments (BSSC and rhythmic slice experiments). Two-way ANOVAs were used to assess the effect of maternal treatment and age (maternal opioids, maternal no treatment) and age (P0-1, P2, P3-5) on respiratory burst frequency or SD1/SD2 (Figures 1, 7). Three-way ANOVAs were used to assess the effect of maternal treatment (maternal opioids, maternal no treatment), age (P0-1, P2, P3-5), and time (baseline average, 120 min average) on percent changes in respiratory activity (Figures 1–3, 6). To correct for multiple comparisons, a Bonferroni post-hoc test was used (*α* = 0.05). No differences were observed between sexes in any *in vitro* electrophysiology experiments (*p* > 0.05); thus, males and females were combined for all *in vitro* electrophysiology experiments. Values are reported as means ± SD and represent biological replicates.

## 3 Results

### 3.1 Litter size, neonatal mortality and neonatal weight gain after maternal opioids

Maternal opioids decreased the number of neonates born per litter (12.8 ± 3.2 neonates after maternal no treatment per litter, 25 litters; 10.6 ± 2.5 neonates after maternal opioids per litter, 23 litters, *p* = 0.03) and increased neonatal mortality at birth (0.6 ± 0.4 neonatal mortality after maternal no treatment, *n* = 215 neonates; 2.1 ± 0.6 neonatal mortality after maternal opioids, *n* = 215 neonates, *p* = 0.04). As shown previously (Hocker et al., 2021), neonates from both maternal groups gained weight significantly each day during development (P0-5), except for P2 to P3 in neonates after maternal no treatment. Between maternal treatment groups, P2 neonates after maternal opioids weighed less than P2 neonates after maternal no treatment (*p* = 0.023), but weight was not significantly different between maternal treatments at any other age (Table 1).

**TABLE 1:** Maternal opioids delayed neonatal weight gain at P2, but not at other ages.

	P0	P1	P2	P3	P4	P5
Maternal opioids	5.1 ± 0.2 g	6.3 ± 0.6 g	7.0 ± 0.4 g^#^	8.1 ± 1.2 g	10.5 ± 1.3 g	11.4 ± 1.0 g
Maternal no treatment	5.2 ± 0.5 g	6.2 ± 0.8 g	8.2 ± 1.1 g	8.3 ± 0.7 g	9.7 ± 1.5 g	11.3 ± 1.2 g

^#^ *p* < 0.05 different from P2 neonates after maternal no treatment; Two-way ANOVA.

### 3.2 Maternal opioids age-dependently impair neonatal central respiratory control networks

To determine the impact of maternal opioids on isolated central respiratory networks, we recorded respiratory-related motor activity from isolated brainstem-spinal cords (BSSCs) containing the neonatal respiratory control network. While maternal treatment and age had no main effect on baseline fictive respiratory burst frequencies (*p* > 0.05), the interaction between maternal treatment and age had a significant main effect on burst frequency (*p* = 0.0085). Pair-wise comparisons showed reduced baseline burst frequencies in P0-1 neonates after maternal opioids (8.3 ± 1.2 burst/min) compared to P0-1 neonates after maternal no treatment (12.2 ± 1.8 burst/min, *p* = 0.03; Figures 1A, B), demonstrating maternal opioids impair central respiratory activity in neonates immediately after birth. These central impairments after maternal opioids were age-dependent, as baseline respiratory burst frequencies were similar in older neonates after maternal opioids (P2 neonates after maternal opioids = 10.8 ± 1.8 burst/min; P3-5 neonates after maternal opioids = 11.8 ± 1.6 burst/min) compared to age-matched neonates after maternal no treatment (P2 neonates after maternal no treatment = 10.5 ± 1.3 burst/min; P3-5 neonates after maternal no treatment = 10.5 ± 2.2 burst/min, *p* >0.9; Figure 1B).

Maternal opioids also significantly impacted burst frequency changes over time. Group analysis demonstrated significant main effects of maternal treatment (*p* = 0.001), time (*p* = 0.0003) and the interaction between treatment and time (*p* = 0.001). No significant main effects were evident on burst frequencies over time with age (*p* = 0.9) or the interactions between maternal treatment and age (*p* = 0.9), age and time (*p* = 0.36), or maternal treatment, age and time (*p* = 0.07). Pairwise comparisons demonstrated burst frequencies decreased from baseline in P2 neonates maternal no treatment (−20 ± 5% change from baseline, *p* = 0.03), while P0-1 neonates after maternal no treatment (−15% ± 10% change from baseline, *p* >0.9) and P3-5 neonates after maternal no treatment (−12 ± 9% change from baseline, *p* >0.9) were unchanged from baseline (Figure 1C). In contrast, burst frequencies decreased from baseline in all neonates after maternal opioids (P0-1 neonates after maternal opioids = −55% ± 34% change from baseline, *p* = 0.01; P2 neonates after maternal opioids = −44 ± 12% change from baseline, *p* = 0.02; P3-5 neonates after maternal opioids = −26 ± 15% change from baseline, *p* = 0.03). Between maternal treatment groups, burst frequencies decreased from baseline in P0-1 neonates after maternal opioids compared to P0-1 neonates after maternal no treatment (*p* = 0.04) and P3-5 neonates after maternal no treatment (*p* = 0.02), but were not different from P2 neonates after maternal no treatment (*p* = 0.2) or older (P2 and P3-5) neonates after maternal opioids (*p* >0.9; Figure 1C). In contrast, respiratory burst frequencies were similar in older neonates (P2 or P3-5) after maternal opioids compared to all neonates after maternal no treatment (*p* > 0.9). Thus, maternal opioids impair neonatal respiratory motor output, demonstrating central respiratory networks within the brainstem and spinal cord are impaired in neonates after maternal opioids.

Similar to changes in frequency, maternal opioids affected neonatal burst amplitude over time (Figure 1D). Maternal treatment (*p* = 0.0092) and time (*p* = 0.0001) and the interaction between maternal treatment and time (*p* = 0.003) had a significant main effect on burst amplitudes over time. Age (*p* = 0.9) or the interactions between maternal treatment and age (*p* = 0.9), age and time (*p* = 0.85), or maternal treatment, age and time (*p* = 0.056) had no significant main effect. After maternal no treatment, burst amplitudes were unchanged from baseline in all neonates after maternal no treatment (P0-1 neonates after maternal no treatment = 8% ± 25% change from baseline, *p* = 0.9; P2 neonates after maternal no treatment = −15 ± 23% change from baseline, *p* = 0.3; P3-5 neonates after maternal no treatment = −26 ± 25% change from baseline, *p* = 0.51). However, burst amplitudes decreased from baseline in all neonates after maternal opioids (P0-1 neonates after maternal opioids = −51% ± 37% change from baseline, *p* = 0.009; P2 neonates after maternal opioids = −36 ± 20% change from baseline, *p* = 0.04; P3-5 neonates after maternal no treatment = −31 ± 19% change from baseline, *p* = 0.048). Thus, maternal opioids impair respiratory activity over time, which further support central impairments in neonatal respiratory networks after maternal opioids, while highlighting a potential role for deficits in respiratory pattern generating nuclei.

Pairwise comparisons between groups showed burst amplitudes decreased from baseline in P0-1 neonates after maternal opioids compared to P0-1 neonates after maternal no treatment (*p* = 0.0287), but burst amplitudes were not different from older (P2 and P3-5) neonates after either maternal treatment (*p* >0.9; Figure 1D). Decreased burst amplitudes were age-dependent, as respiratory burst amplitudes were similar in older neonates (P2 or P3-5) after maternal opioids compared to age-matched neonates after maternal no treatment (*p* > 0.9). Thus, maternal opioids age-dependently impair neonatal central respiratory network activity, similar to destabilized breathing in neonates after maternal opioids (Hocker et al., 2021).

### 3.3 Lingering neonatal opioids contribute to impaired central respiratory network activity in neonates after maternal opioids

To investigate the contribution of continued opioid receptor activation to decreasing central respiratory activity in neonates after maternal opioids, we examined respiratory activity from isolated neonatal BSSCs in the presence of a mu-opioid receptor antagonist (naloxone, 10 µM). Group data demonstrated no main effects of any factor (maternal treatment, age, time or the interactions between the three factors) on burst frequency over time after mu-opioid receptor antagonism (*p* > 0.9) and no pairwise differences in any maternal treatment or age (*p* >0.9; Figures 2A, B). Thus, reduced baseline respiratory burst frequencies in P0-1 neonates after maternal opioids are unlikely to be due to continued neonatal opioid receptor activation.

While opioid receptor antagonism did not significantly impact burst frequencies over time, it had a significant effect on burst amplitudes over time. Maternal treatment (*p* = 0.0001), age (*p* = 0.0001), time (*p* = 0.01), and interactions between treatment and age (*p* = 0.0001), maternal treatment and time (*p* = 0.0001), and maternal treatment, age and time (*p* = 0.001) had significant main effects on burst amplitude over time. In neonates after maternal no treatment, burst amplitudes decreased from baseline after mu-opioid receptor antagonism in P2 neonates after maternal no treatment (−17% ± 5% change from baseline, *p* = 0.04), while P0-1 neonates after maternal no treatment (−14% ± 4% change from baseline, *p* = 0.06) and P3-5 neonates after maternal no treatment (−12% ± 4% change from baseline, *p* = 0.7) were unchanged from baseline (Figure 2C). In contrast, burst amplitudes after mu-opioid receptor antagonism increased from baseline in P0-1 neonates after maternal opioids (14% ± 6% change from baseline, *p* = 0.03), while burst amplitude from older neonates after maternal opioids were unchanged from baseline (P2 neonates after maternal opioids = −10% ± 6% change from baseline, *p* = 0.09; P3-5 neonates after maternal opioids = −12% ± 4% change from baseline, *p* = 0.07). The decrease in burst frequency and amplitude in neonates after maternal no treatment is consistent with previous studies using *in vitro* electrophysiology to study the respiratory network activity (Alvares et al., 2014; Blitz and Ramirez, 2002; Camacho-Hernández et al., 2022; Johnson et al., 2012), reflecting typical changes in activity over time. Thus, lingering neonatal opioids may contribute to acute changes in burst amplitude, but not frequency, in neonates after maternal opioids.

Pairwise comparisons between groups demonstrated burst amplitudes increased over time after mu-opioid receptor antagonism in P0-1 neonates after maternal opioids compared to all neonates after maternal no treatment (P0-1: *p* = 0.0001; P2: *p* = 0.0002; P3-5: *p* = 0.0001) and older (P2 and P3-5) neonates after maternal opioids (P2: *p* = 0.0009; P3-5: *p* = 0.0004; Figure 2C). The effect of mu-opioid receptor antagonism on central respiratory activity in neonates after maternal opioids was age-dependent, as burst amplitudes over time were similar in older (P2 and P3-5) neonates after maternal opioids compared to all neonates after maternal no treatment (*p* > 0.9). These acute increases in amplitude in response to opioid receptor antagonism suggest lingering opioids impact central nuclei generating or modulating respiratory pattern in neonates after maternal opioids.

### 3.4 Maternal opioids blunted neonatal central respiratory responses to exogenous opioid immediately after birth

To determine the contribution of opioid receptors to central respiratory responses to exogenous opioids after maternal opioids, we recorded respiratory activity in isolated BSSCs after mu-opioid receptor agonism (methadone, 10 µM). Maternal opioids caused inter-preparation variability in the acute responses to exogenous opioids. Respiratory activity was maintained after acute opioids in younger neonates after maternal opioids (P0-1: 5/6 experiments; P2: 3/6 experiments), while activity was not maintained in older neonates after maternal opioids (P3-5: 0/4 experiments) nor neonates after maternal no treatment (P0-1: 0/6 experiments; P2: 0/5 experiments; P3-5: 1/6 experiments).

There was a main effect of maternal treatment (*p* = 0.0005), age (*p* = 0.0098), time (*p* = 0.002), and the interactions between maternal treatment and age (*p* = 0.0035), maternal treatment and time (*p* = 0.009), age and time (*p* = 0.006), and maternal treatment, age, and time (*p* = 0.002) on respiratory burst frequency after mu-opioid receptor agonism. As expected, exogenous opioids abolished burst frequency in neonates after maternal no treatment at all ages (P0-1 neonates after maternal no treatment = −100% ± 0% change from baseline, *p* = 0.001; P2 neonates after maternal no treatment = -100% ± 0% change from baseline, *p* = 0.001; P3-5 neonates after maternal no treatment = −97% ± 7% change from baseline, *p* = 0.001; Figures 3A, B). In neonates after maternal opioids, burst frequencies decreased from baseline, but were not abolished, after exogenous opioids in P0-1 and P2 neonates (P0-1 neonates after maternal opioids = −48% ± 26% change from baseline, *p* = 0.006; P2 neonates after maternal opioids = −77% ± 25% change from baseline, *p* = 0.002). Activity was abolished in older neonates after acute opioids (P3-5 neonates after maternal opioids = -100% ± 0% change from baseline; *p* = 0.001; Figure 3B). Importantly, pairwise comparisons between groups showed burst frequencies were maintained in P0-1 neonates after maternal opioids compared to all neonates after maternal no treatment (*p* < 0.05) and P3-5 neonates after maternal opioids (*p* = 0.03), but were similar to P2 neonates after maternal opioids (*p* = 0.8; Figure 3B). Thus, maternal opioids blunt central respiratory responses to exogenous opioids. Blunted central responses to exogenous opioids are age-dependent, as decreases in burst frequency in older neonates after maternal opioids (P2 and P3-5) were not significantly different from neonates after maternal no treatment at any age (*p* = 0.9; Figure 3B).

Similar to changes in burst frequency, burst amplitude was age-dependently blunted by exogenous opioids (Figures 3A, C). Maternal treatment (*p* = 0.002), time (*p* = 0.0001), and the interactions between maternal treatment and time (*p* = 0.002), maternal treatment and age (*p* = 0.008), and maternal treatment, age, and time (*p* = 0.008) had significant main effects on burst amplitude after mu-opioid receptor agonism, with no main effect of age (*p* = 0.055) or the interaction between age and time (*p* = 0.96). As expected, exogenous opioids abolished burst amplitude in neonates after maternal no treatment at all ages (P0-1 neonates after maternal no treatment = −100% ± 0% change from baseline, *p* = 0.0001; P2 neonates after maternal no treatment = −100% ± 0% change from baseline, *p* = 0.0001; P3-5 neonates after maternal no treatment = −93% ± 16% change from baseline, *p* = 0.0002; Figure 3C). Similarly, burst amplitude decreased from baseline after exogenous opioids in all neonates after maternal opioids (P0-1 neonates after maternal opioids = −46% ± 26% change from baseline, *p* = 0.005; P2 neonates after opioids = −72% ± 31% change from baseline, *p* = 0.003; P3-5 neonates after opioids = −93% ± 16% change from baseline, *p* = 0.0002; Figure 3C). Pairwise comparisons between groups demonstrated burst amplitudes were maintained over time after exogenous opioids in P0-1 neonates after maternal opioids compared to all neonates after maternal no treatment (*p* < 0.05) and P3-5 neonates after maternal opioids (*p* = 0.01), but were similar to P2 neonates after maternal opioids (*p* = 0.9; Figure 3C). Blunted central responses to exogenous opioids were age-dependent, as exogenous opioids abolished burst amplitude in older (P2 and P3-5) neonates after maternal opioids, similar to all neonates after maternal no treatment (*p* = 0.9; Figure 3C). Thus, respiratory activity (frequency and amplitude) after exogenous opioids was age-dependently maintained in neonates after maternal opioids, supporting acute protection from significant opioid-induced respiratory depression immediately after birth (Hocker et al., 2021).

Since changes in respiratory activity over time failed to capture the dynamic changes in activity in response to acute opioids, we assessed the probability of maintaining activity using survival curves. The probability of maintaining respiratory activity after mu-opioid receptor agonism was significantly higher in P0-1 and P2 neonates after maternal opioids compared to all neonates after maternal no treatment (*p* < 0.001) and P3-5 neonates after maternal opioids (*p* < 0.001; Figure 3D). Thus, central respiratory networks from P0-1 and P2 neonates after maternal opioids are less responsive to exogenous opioids, likely contributing to blunted opioid-induced respiratory depression in neonates after maternal opioids (Hocker et al., 2021).

### 3.5 Mu-opioid receptor immunoreactivity in the preBötzinger Complex qualitatively decreased during postnatal development

**FIGURE 4:**
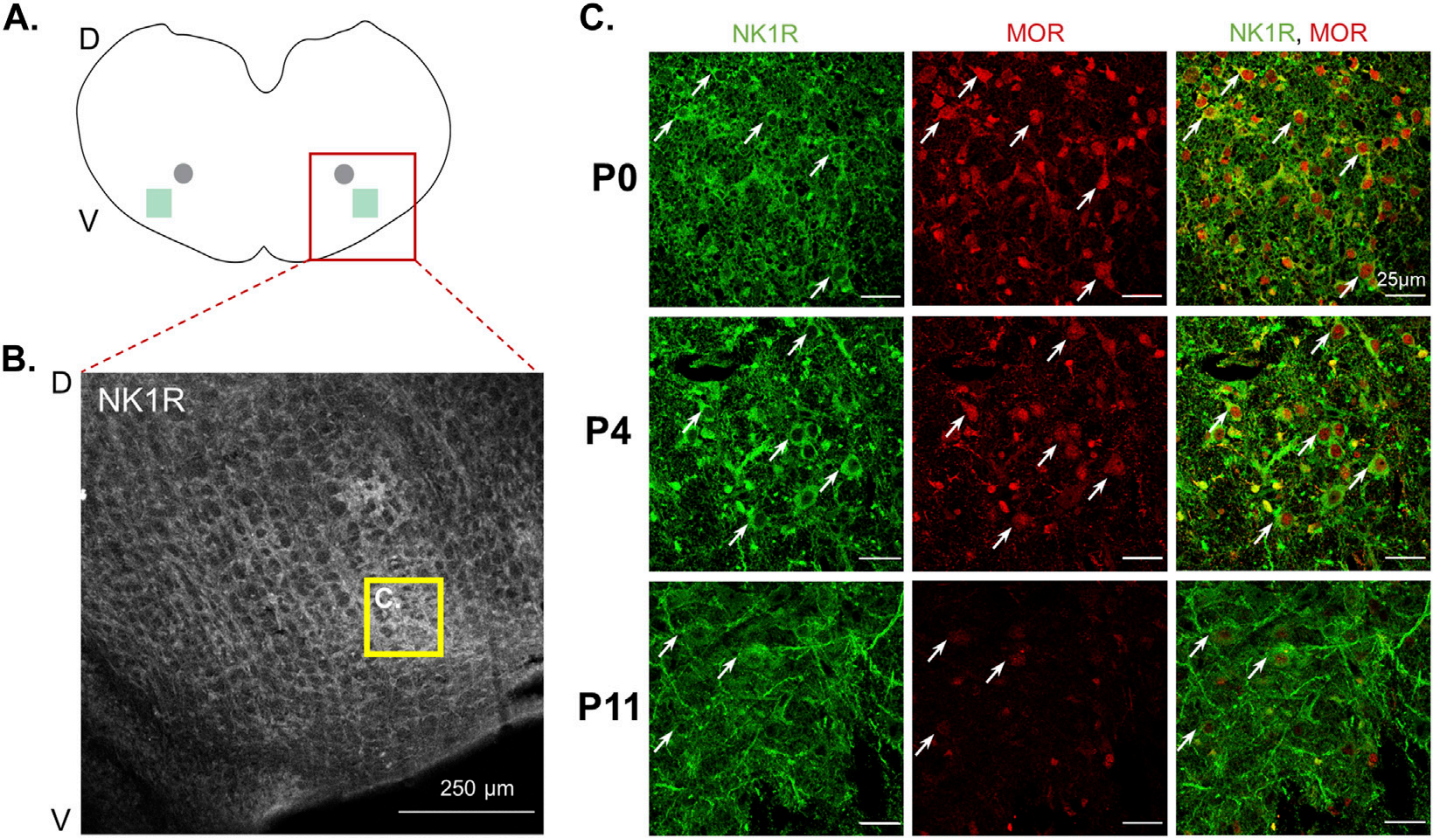
Mu-opioid receptor expression decreased in neonatal preBötzinger complex NK1R + neurons during postnatal development. Schematic of transverse medullary slices **(A)** highlights the preBötzinger Complex (preBötC, green square) located lateral and ventral to the semi-compact nucleus ambiguous (gray circle). Representative confocal images [**(B)**, 10x] identify neurokinin-1 receptor positive (NK1R+) neurons in the preBötC (yellow box). Representative confocal images [**(C)**, 40x] demonstrate NK1R + preBötC neurons (green) express mu-opioid receptors (MOR) at all ages; however, P0 neonates appear to have the strongest expression of mu-opioid receptor in NK1R + preBötC neurons (white arrows) compared to P4 neonates and mu-opioid receptor appeared to have the lowest expression in P11 neonates (*n* = 6, 3 males, 3 females per age group).

Results from opioid receptor antagonist and agonist experiments support a role for opioid receptors in changing respiratory activity in neonates after maternal opioids, highlighting the need to understand opioid receptor expression in neonatal central respiratory control networks. Further, decreased burst frequency in P0-1 neonates after maternal opioids also suggests the preBötzinger Complex (preBötC) may contribute to central impairments in respiratory networks. Thus, we investigated the effect of maternal opioids on mu-opioid receptor expression in an essential respiratory rhythm generating center, the preBötC. First, we characterized typical mu-opioid receptor expression in the preBötC during postnatal development (P0, P4, P11). Using immunohistochemistry, mu-opioid receptors and neurokinin-1 receptor positive (NK1R+) neurons were labeled in the ventral lateral medulla. Using anatomical landmarks, the preBötC was identified as a fusiform cluster of neurons ventral and lateral from nucleus ambiguous (Figures 4A, B), based on previous work (Gray et al., 1999; Pagliardini et al., 2003; Huxtable et al., 2010). During typical postnatal development, mu-opioid receptor immunoreactivity appeared highest immediately after birth (P0) and decreased during postnatal development (Figure 4C). Additionally, mu-opioid receptor immunoreactivity on NK1R + preBötC neurons at P4 appeared greater than in P11 neonates (Figure 4C), supporting a progressive decrease in mu-opioid receptors during typical postnatal development.

### 3.6 Maternal opioids qualitatively decreased opioid receptor expression on neonatal preBötzinger Complex neurons

**FIGURE 5:**
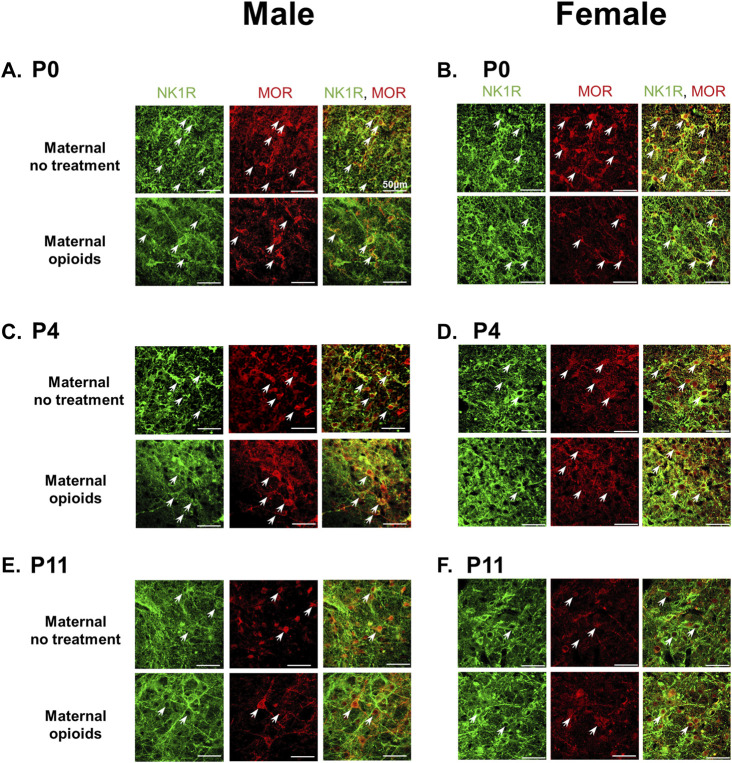
Mu-opioid receptor expression on preBötzinger Complex NK1R + neurons decreased in P0 neonates after maternal opioids, but not at P4 or P11. Representative confocal images (40x) show NK1R + preBötzinger Complex neurons (green) and mu-opioid receptors (red) colocalization (white arrows) decreased in P0 male **(A)** and female **(B)** neonates after maternal opioids compared to P0 sex-matched neonates after maternal no treatment. Mu-opioid receptors were similar in P4 male **(C)** and female **(D)** neonates after maternal opioids and P11 male **(E)** and female **(F)** neonates after maternal opioids compared to age- and sex-matched neonates after maternal no treatment (*n* = 3 per treatment, age and sex; scale bars 50 μm).

After establishing the typical expression of mu-opioid receptors in the preBötC during postnatal development, we used immunohistochemistry on a separate group of neonates to determine the effect of maternal opioids on mu-opioid receptors in the neonatal preBötC. Male and female P0 neonates after maternal opioids appeared to have less mu-opioid receptor immunoreactivity in NK1R + neurons compared to P0 neonates after maternal no treatment (Figures 5A, B). However, male and female P4 and P11 neonates after maternal opioids had similar immunoreactivity to age-matched neonates after maternal no treatment (Figures 5C–F). The immunohistochemistry results are consistent with changes in respiratory activity and further support similarities between the sexes after maternal opioids. Thus, opioid receptor expression decreases with typical postnatal development and decreases immediately after birth following maternal opioids.

### 3.7 Respiratory activity in neonatal medullary rhythmic slices were unaffected by maternal opioids

**FIGURE 6:**
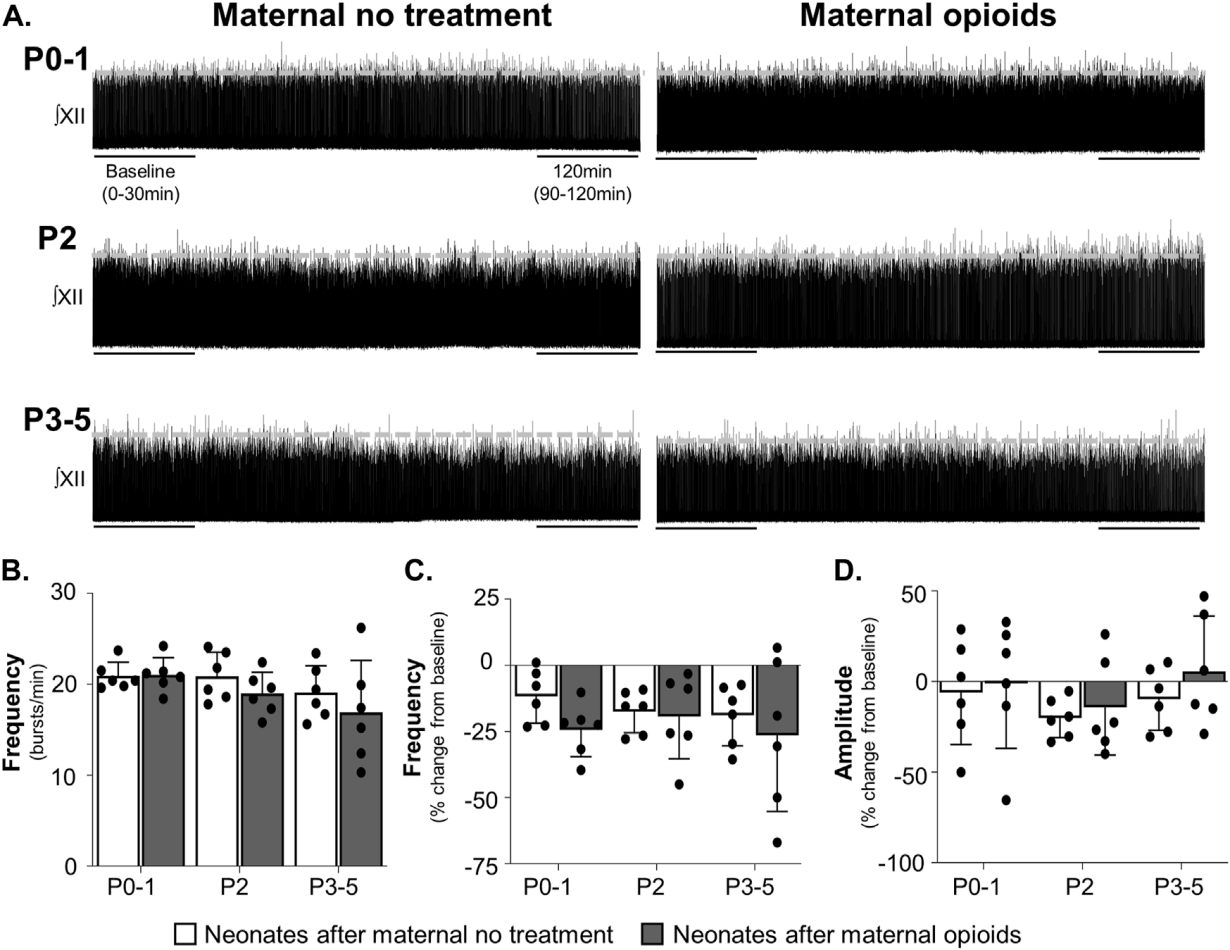
Isolated respiratory network activity was maintained in rhythmically active slices containing the preBötzinger Complex in neonates after maternal opioids. Representative neurograms **(A)** show respiratory activity from isolated rhythmic slices was maintained in neonates after maternal opioids at all ages. Baseline neonatal burst frequencies **(B)** were similar in neonates, regardless of maternal treatment or age. Over time, isolated rhythmic slice burst frequencies **(C)** and amplitudes **(D)** were unchanged from baseline and similar between maternal treatments and ages (Three-way ANOVA; Bonferroni post-hoc test).

To identify where within the brainstem-spinal cord networks maternal opioids impair respiratory activity, we utilized a further reduced preparation, consisting of the preBötC, hypoglossal motor nucleus, and hypoglossal nerve roots (Greer et al., 1991; Smith et al., 1991). In contrast to respiratory activity from BSSCs, maternal treatment, age or the interaction between maternal treatment and age did not have a significant main effect on baseline respiratory burst frequencies (*p* > 0.9). Baseline burst frequencies were similar regardless of maternal treatment or age (P0-1 neonates after opioids = 21 ± 2 burst/min; P2 neonates after opioids = 19 ± 2 burst/min; P3-5 neonates after opioids = 17 ± 5 burst/min; P0-1 neonates after maternal no treatment = 21 ± 1 burst/min; P2 neonates after maternal no treatment = 21 ± 3 burst/min; P3-5 neonates after maternal no treatment = 19 ± 3 burst/min, *p* > 0.1; Figures 6A, B).

Maternal treatment also did not change burst frequency or amplitude over time. Neither maternal treatment, age, time, nor the interactions between the three factors had a significant main effect on respiratory burst frequencies over time (*p* > 0.9). No pairwise differences exist in isolated medullary burst frequencies over time (P0-1 neonates after maternal no treatment = −11% ± 9% change from baseline, *p* = 0.3; P2 neonates after maternal no treatment = −18% ± 7% change from baseline, *p* = 0.08; P3-5 neonates after maternal no treatment = −19% ± 11% change from baseline, *p* = 0.07; P0-1 neonates after maternal opioids = −24% ± 9% change from baseline, *p* = 0.07; P2 neonates after maternal opioids = −19% ± 15% change from baseline, *p* = 0.08; P3-5 neonates after maternal opioids = −26% ± 26% change from baseline, *p* = 0.07; Figure 6C).

Similarly, neither maternal treatment, age, time, or interactions between the three factors had significant main effects on neonatal burst amplitudes over time (*p* > 0.9), with no pairwise differences (P0-1 neonates after opioids = −1 ± 32% change from baseline, *p* = 0.9; P2 neonates after opioids = −14% ± 24% change from baseline, *p* = 0.09; P3-5 neonates after opioids = 6% ± 28% change from baseline *p* = 0.09; P0-1 neonates after maternal no treatment = −6% ± 26% change from baseline, *p* = 0.8; P2 neonates after maternal no treatment = −20% ± 10% change from baseline, *p* = 0.07; P3-5 neonates after maternal no treatment = −10% ± 16% change from baseline, *p* = 0.1; Figure 6D). In summary, these data suggest central impairments in respiratory networks in P0-1 neonates after maternal opioids extend beyond the preBötC.

### 3.8 Maternal opioids impair neonatal respiratory pattern in isolated brainstem-spinal cords, but not medullary rhythmic slices, at all ages

**FIGURE 7:**
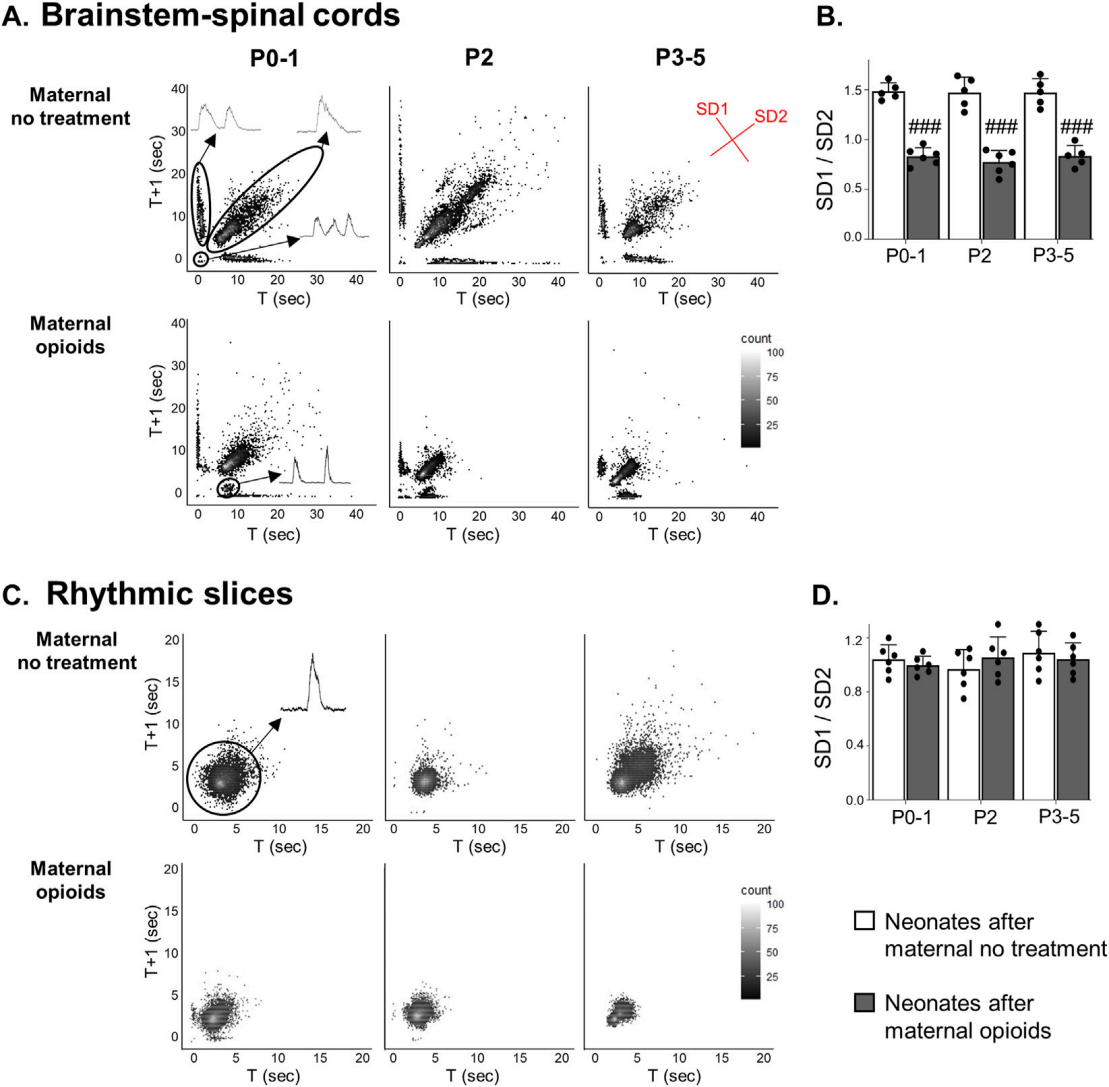
A distinct respiratory pattern emerged in neonatal isolated brainstem-spinal cords, but not rhythmic slices, after maternal opioids. Poincaré plots **(A)** show burst-to-burst variation in isolated brainstem-spinal cords (BSSCs). Three respiratory burst patterns were prominent after maternal no treatment: singlets, doublets, and triplets (inset). In neonates after maternal opioids, a distinct respiratory burst pattern emerged. SD1/SD2 measurements of burst-to-burst variation in isolated BSSCs **(B)** decreased in neonates after maternal opioids compared to neonates after maternal no treatment. In rhythmically active slices, singlet burst activity was prominent (inset) in all treatment groups and ages **(C)** and burst-to-burst variation **(D)** was unchanged in any treatment group or age (^###^ *p* < 0.0001 different from neonates maternal no treatment at all ages; Two-way ANOVA; Bonferroni post-hoc test).

Assessments of frequency and amplitude failed to capture intricate changes in respiratory burst pattern. Thus, to better describe the effects of maternal opioids on neonatal respiratory burst pattern, Poincaré plots were generated from neonatal respiratory activity in isolated brainstem-spinal cords (BSSCs) and medullary rhythmic slices. In isolated BSSCs, neonates after maternal no treatment displayed three respiratory burst patterns: singlets, doublets, and triplets (Figure 7A, top panels). However, neonates after maternal opioids exhibited a unique respiratory pattern, a singlet burst with a decreased period to the next burst (Figure 7A, bottom panels). When quantifying overall burst-to-burst variability with SD1/SD2, there was a main effect of maternal treatment (*p* = 0.0001), but not age (*p* = 0.9) or the interaction between maternal treatment and age (*p* = 0.9), in respiratory activity from isolated BSSCs. Burst-to-burst variation in respiratory activity from isolated BSSCs was lower in all neonates after maternal opioids (SD1/SD2: P0-1 neonates after opioids = 0.78 ± 0.12; P2 neonates after opioids = 0.8 ± 0.13; P3-5 neonates after opioids = 0.82 ± 0.1) compared to all neonates after maternal no treatment (SD1/SD2: P0-1 neonates after maternal no treatment = 1.5 ± 0.12; P2 neonates after maternal no treatment = 1.4 ± 0.18; P3-5 neonates after maternal no treatment = 1.6 ± 0.13, *p* < 0.0001; Figure 7B).

In contrast to respiratory activity from isolated BSSCs, rhythmic slices exhibited regular singlet patterns (Figure 7C). SD1/SD2 burst-to-burst variation of respiratory activity in medullary rhythmic slices was unchanged in all neonates after maternal opioids compared to all neonates after maternal no treatment (*p* > 0.9; Figure 7D). Maternal treatment, age or the interaction between maternal treatment and age (*p* > 0.9) had no a main effect on burst-to-burst variation from medullary rhythmically active slices and there were no pairwise differences between maternal treatment or age (SD1/SD2: P0-1 neonates after opioids = 1 ± 0.1; P2 neonates after opioids = 0.96 ± 0.13; P3-5 neonates after opioids = 1.1 ± 0.14; P0-1 neonates after maternal no treatment = 1 ± 0.06; P2 neonates after maternal no treatment = 1.1 ± 0.14; P3-5 neonates after maternal no treatment = 1 ± 0.11, *p* >0.9; Figure 7D). Thus, a diverse pattern of activity appears with more complete respiratory network circuitry and maternal opioids evoked a distinct pattern of a single burst with a decreased period to the next burst, not evident in more isolated respiratory networks, supporting maternal opioids may be impairing respiratory circuity beyond the preBötC networks.

## 4 Discussion

A clinical population of infants with NAS is continuing to grow (Krans, et al., 2015; Pryor et al., 2017) and these infants experience respiratory deficits (Fältmarch et al., 2019), highlighting a need to understand the etiology of breathing deficits in these infants. Here, we capitalize on our previous characterizations of neonatal breathing deficits after late gestational maternal opioids (Hocker et al., 2021) and target central deficits in neonates after maternal opioids. Since breathing is controlled centrally by neural networks in the brainstem and spinal cord (reviewed in Del Negro et al., 2018), we began by studying the neural impairments in progressively more isolated central respiratory networks. We demonstrate maternal opioids age-dependently impair central respiratory networks; however, the deficits include regions beyond the preBötzinger Complex (preBötC), specifically those associated with respiratory pattern. Lingering opioids in neonatal central respiratory networks contribute to respiratory amplitude deficits immediately after birth, but not frequency. Since opioids likely remain in the neonatal brain for multiple days after maternal opioid exposure (Farid et al., 2008; Kongstorp et al., 2019), lingering neonatal opioids are likely of maternal origin. Yet, lingering opioids do not account for decreased respiratory frequency or blunted opioid-induced respiratory depression immediately after birth. Blunted respiratory responses to acute, exogenous opioids may, in part, be explained by decreased mu-opioid receptors in the preBötC, supporting diverse impairments after maternal opioids. Collectively, this study provides novel insights into the direct, central effects of maternal opioids on the neonatal respiratory system, further advancing our understanding the respiratory deficits in infants with NAS.

The respiratory network must be functional at birth to maintain homeostasis, but the respiratory network continues to mature postnatally (Patrone et al., 2018; Patrone et al., 2020; Taxini et al., 2021; reviewed in Wong-Riley et al., 2019), including refinement of respiratory rhythm generation (Bissonnette et al., 1991; Kobayashi et al., 2001), shifts in oscillator dominance (Onimaru and Dutschmann, 2012; Hocker et al., 2021), and shifts in neurotransmitter/neuromodulator receptor expression (Liu and Wong-Riley, 2002; Gao et al., 2011; Wong-Riley et al., 2013). While lingering neonatal opioids contribute to reductions in respiratory amplitude in neonates after maternal opioids, we hypothesize that reduced respiratory frequency in neonates after maternal opioids is caused by disruption of perinatal maturation of respiratory networks after maternal opioids. We previously identified quantal slowing emerged at P2 in neonates, suggesting P2 is a key developmental age for shifting oscillator dominance (Hocker et al., 2021). In support of this, central impairments in isolated neonatal respiratory control networks were most prominent in young neonates (P0-2), whereby older neonates demonstrated more rhythmic, consistent central activity. Interestingly, deficits in respiratory pattern continued in older neonatal ages, suggesting a potential for lasting impairment of respiratory networks beyond the deficits in breathing. None of these changes in respiratory activity were evident in neonates after maternal no treatment, with the exception of a decrease in activity over time acutely at P2. In fact, this finding may further support increased vulnerability during this window of network reorganization at P2 and decrease longevity of respiratory activity *in vitro*. Thus, our research provides insights into how maternal opioids impair perinatal maturation of the respiratory network, which is key to understanding the etiology of breathing deficits in neonates after maternal opioids (Hocker et al., 2021).

Central respiratory networks consist of a diverse network of cells within the brainstem, whereby the medulla is the primary site for respiratory rhythm generation (reviewed in Del Negro et al., 2018). Essential neurons within these networks are born earlier in gestation (E10-E14), yet they remain immature even at birth (Pagliardini et al., 2003; Gray et al., 2010; Revill et al., 2015; Akins et al., 2017; Kottick et al., 2017). The deficits here after late gestation maternal opioids were investigated in medullary networks, whereby maternal opioids directly impaired central medullary network activity. Deficits, however, extended beyond impairments to the preBötC and are consistent with previous research showing maternal opioids induce reorganization of neonatal respiratory control networks (Gourévitch et al., 2016). Increased amplitude and emergence of a distinct respiratory pattern support actions on opioid-sensitive respiratory regions generating and/or modulating respiratory burst pattern, such as the opioid-sensitive Post-Inspiratory Complex (Ramirez and Baertsch, 2018), the Bötzinger Complex (Cinelli et al., 2020), premotor neurons (i.e., rostral ventral respiratory group; Lonergan et al., 2003), motoneurons (i.e., phrenic motor neurons; Xia and Haddad, 1991), the opioid-insensitive parafacial respiratory group/retrotrapezoid nucleus (pFRG/RTN; Onimaru et al., 1997; Onimaru and Homma, 2003; 2005; Onimaru and Dutschmann, 2012) and other modulatory regions (i.e., the nucleus tractus solitarius, NTS; Endoh, 2006; or the caudal medullary raphe neurons; Palkovic et al., 2022). Although pontine regions, such as the Kolliker-Fuse and the parabrachial complex, are opioid-sensitive and contribute to opioid-induced respiratory depression (Baldo, 2022; Bateman et al., 2021; Prkic et al., 2012; Saunders and Levitt, 2020), these regions do not contribute to central respiratory network impairments observed here, as the pons was removed. Findings from this study provide potential avenues for future investigation and suggest the importance of further analyses on respiratory pattern. Thus, maternal opioids directly impair central neonatal respiratory networks late in gestation after initial establishment of vital respiratory circuitry (Pagliardini et al., 2003; Ren and Greer, 2003; Greer, 2015).

Respiratory burst pattern is regulated by multiple distinct respiratory regions throughout the medulla and spinal cord, leading to the generation of inspiratory, post-inspiratory and pre-inspiratory neural activity; all of which contribute to neonatal breathing at rest (reviewed in Del Negro et al., 2018). Here, we identified a distinct respiratory burst pattern of a single burst with a decreased period to the next burst in neonates after maternal opioids at all ages, suggesting maternal opioids may cause lasting alterations to neonatal respiratory pattern. After maternal opioids, the distinct burst pattern occurred in more complete respiratory networks (brainstem and spinal cords), but not in more isolated networks (medullary slices containing the preBötzinger Complex), suggesting changes in neonatal respiratory pattern extend beyond the preBötC. Three possibilities could explain the distinct respiratory pattern in neonates after maternal opioids. First, maternal opioids may reduce post-inspiratory activity from opioid-sensitive PiCo neurons (Anderson et al., 2016; reviewed in Ramirez and Baertsch, 2018) or impair recurrent excitatory or inhibitory connections between preBötC and PiCo neurons coordinating respiratory burst timing (reviewed in Ramirez and Baertsch, 2018). Second, a mismatch in pre-inspiratory activity from opioid-insensitive pFRG/RTN neurons and inspiratory activity from the opioid-sensitive preBötC neurons may contribute to the distinct respiratory burst pattern in neonates after maternal opioids. Third, maternal opioids may decrease activity in the opioid-sensitive NTS (Endoh, 2006) or disrupt excitatory or inhibitory recurrent connections between the NTS and preBötC (Yang et al., 2020) to influence respiratory burst pattern in neonates after maternal opioids. Breathing normalized in older neonates after maternal opioids (Hocker et al., 2021); however, the distinct respiratory pattern occurred at all neonatal ages, suggesting the change in respiratory pattern is a lasting change in respiratory networks.

The distinct respiratory pattern induced by maternal opioids was similar to a pattern observed after neonatal inflammation (Morrison et al., 2019). Although TLR4 signaling activated by neonatal inflammation does not contribute to acute opioid-induced respiratory depression in neonates (Zwicker et al., 2014), significant cross-talk between opioid receptors and inflammatory signaling pathways exists (reviewed in Zhang et al., 2020), which may explain some similarities in responses. In fact, perinatal opioid exposure increases neonatal cortical chemokines and cytokines, alters microglial morphology and upregulates cortical microglial TLR4 and MyD88 RNA expression (Jantzie et al., 2020). Thus, it remains possible that maternal opioids alter inflammatory signaling in neonatal respiratory networks to induce changes in neural activity.

Our results demonstrating maternal opioids decrease respiratory burst amplitude and emergence of a distinct respiratory pattern at all ages provide support for the hypothesis that maternal opioids disrupt perinatal maturation of central respiratory networks. Many early life stressors, such as gestational intermittent hypoxia (Gozal et al., 2003; Johnson et al., 2017), gestational ethanol exposure (Dubois and Pierrefiche, 2020), prenatal anxiety drug exposure (da Silva Junior et al., 2022), perinatal inflammation (Morrison et al., 2019; Camacho-Hernández et al., 2022), perinatal nicotine exposure (Luo et al., 2004; Ferng and Fregosi, 2015; Cholanian et al., 2017), perinatal anti-depressant drug exposure (Biancardi et al., 2022) and neonatal maternal separation (Kinkead et al., 2005; Kinkead and Gulemetova, 2010; Rousseau et al., 2017; reviewed in Tenorio-Lopes and Kinkead, 2021), acutely disrupt neonatal respiratory control. Some of these stressors even have enduring consequences on the adult respiratory system (Genest et al., 2004; Kinkead et al., 2009; Soliz et al., 2016; Hocker et al., 2019; Dubois and Pierrefiche, 2020; Biancardi et al., 2022). This research supports maternal opioids as a significant early life stressor, despite aspects of neonatal breathing normalizing over time after maternal opioids and has the potential to induce lasting impairments on central respiratory networks into adulthood.

Additional exogenous opioids are frequently given to human infants with NAS to curb withdrawal symptoms (Jansson and Velez, 2012), yet the impact of additional exogenous opioids on central respiratory networks has not been studied. Similar to the effects we observed with neonatal breathing (Hocker et al., 2021), responses of isolated central networks to exogenous opioids in neonates after maternal opioids were acutely blunted and correlate with decreased opioid receptor expression. Also consistent with previous research (Hocker et al., 2021), mu-opioid receptor expression was similar between P4 neonates after maternal no treatment and maternal opioids, when breathing normalized after maternal opioids, despite neonates continuing to receive opioids through breast milk (Naumburg and Meny, 1988; Ito, 2018). Future research is needed to determine the mechanisms for normalized responses to exogenous opioids in older neonates (P3-5), despite this continued exposure to opioids. There were no lasting or rebound effects of ceasing maternal opioid exposure since mu-opioid receptor expression was similar at P11, after maternal opioid exposure ceased. These observations are consistent with those from the locus coeruleus, showing opioid receptor expression decreased during chronic adult opioid exposure and returned to pre-exposure levels after opioid exposure ceased (Quillinan et al., 2011; Dang et al., 2012). Developmental changes in responses to exogenous opioids (evident in survival curves) is also consistent with clinical observations that infants with NAS have highly variable responses to additional opioids in the first week of life (Jansson and Velez, 2012). In summary, our research begins to investigate central mechanisms likely contributing to blunted opioid-induced respiratory depression in neonates after maternal opioids (Hocker et al., 2021), which is needed to understand opioid sensitivities in infants with NAS during the first week of life (Jansson and Velez, 2012).

Many different central respiratory regions are involved in opioid-induced respiratory depression (Greer et al., 1995; Mellen et al., 2003; Pattinson, 2008; Bell et al., 2011; Montandon et al., 2011; Emery et al., 2016; Bachmutsky et al., 2020; Varga et al., 2020; Baertsch et al., 2021; Bateman et al., 2021) and may contribute to blunted respiratory responses to exogenous opioids in neonates after maternal opioids. Candidate respiratory regions for future investigation of blunted central responses to exogenous opioids in neonates after maternal opioids include: the opioid-sensitive rostral ventral respiratory group (Cinelli et al., 2020; Lonergan et al., 2003; Miyawaki et al., 2002), the Bötzinger Complex (Cinelli et al., 2020), and the caudal medullary raphe (Palkovic et al., 2022) because these regions significantly contribute to acute opioid-induced respiratory depression. The opioid-insensitive pFRG/RTN may also contribute to blunted opioid-induced respiratory depression, since the pFRG/RTN is the dominant respiratory rhythm generator immediately after birth (Onimaru et al., 1989; Onimaru et al., 1995; Onimaru and Dutschmann, 2012; Hocker et al., 2021). Maternal opioids may then increase reliance on the pFRG/RTN to maintain respiratory activity despite the ongoing presence of additional opioids, either of maternal origin or in response to additional exogenous opioids used to treat withdrawal symptoms (Kraft et al., 2016; Mangat et al., 2019). Understanding specific regions in the respiratory network contributing to blunted neural and breathing responses to exogenous opioids in neonates after maternal opioids is needed to determine the potentially unique mechanisms underlying opioid-induced respiratory depression in neonates after maternal opioids.

Significant research efforts are focused on how opioids depress respiratory control networks, primarily in adults (Greer et al., 1995; Takita et al., 1997; Stucke et al., 2008; 2015; Bell et al., 2011; Montandon et al., 2011; 2016; Boom et al., 2012; Prkic et al., 2012; Saunders and Levitt, 2020; Bateman et al., 2021; Palkovic et al., 2022); yet, less is known about postnatal development of opioid-induced respiratory depression and changes in opioid receptor expression. Early studies investigated opioid receptor expression in the brainstem during postnatal development used quantitative receptor autoradiography, showing opioids bind to many neonatal medullary respiratory regions, such as dorsal parabrachial nucleus, NTS, Kölliker-Fuse nucleus, and the ventral parabrachial nucleus (Xia and Haddad, 1991). Opioid receptors in many respiratory control regions increased with age, until maximal expression between P10 and P21 before decreasing in adulthood (Xia and Haddad, 1991). Others identified mu-opioid receptor expression in a subset of NK1R positive preBötC neurons (Gray et al., 1999; Pagliardini et al., 2003; Montandon et al., 2011; Cinelli et al., 2020), yet the expression of mu-opioid receptors in the preBötC during early postnatal development remained unclear. Here, we characterized the expression of mu-opioid receptors in the putative preBötC during early postnatal development, demonstrating mu-opioid receptors appear highest at P0, decrease at P4, and decrease further from P4 to P11. This provides region-specific insights into opioid receptor expression in the preBötC across postnatal development and suggests neonatal breathing responses to exogenous opioids may shift during typical postnatal development as opioid receptor expression changes.

The type, dose, and dosing paradigm of opioids used to study the developmental effects of opioids remain controversial and complicated (reviewed in Farid et al., 2008). This is further complicated by species-specific differences in tolerance, withdrawal, and stress (reviewed in Farid et al., 2008). As such, the dose of maternal opioids used here (5 mg/kg methadone, s.c, daily G17-P5) may lead to lower fetal and neonatal concentrations of opioids than human fetuses and infants exposed to maternal opioids (De Montis et al., 1983). It remains challenging to replicate the complex and extenuating factors influencing fetal opioid exposure in humans (Farid et al., 2008). However, substantial other work in rodent models support the dose of methadone used here (reviewed in Farid et al., 2008) and we have previously shown this dose paradigm impaired neonatal breathing (Hocker et al., 2021). Future work should explore the effects of different opioids at varying doses and developmental times to further mimic the complexity of NAS.

In conclusion, this research study provides insights into the etiology of breathing deficits in infants with NAS, by determining the direct effects of maternal opioids on central neonatal respiratory networks within the brainstem and spinal cord. Maternal opioids age-dependently disrupted neonatal respiratory network activity and circulating neonatal opioids contributed to central respiratory impairments in neonates after maternal opioids. Interestingly, central impairments extend beyond the preBötC and involve changes in respiratory pattern. Such central impairments in neonatal respiratory control after maternal opioids likely contribute to destabilized breathing (Hocker et al., 2021); however, maternal opioids blunt central responses to exogenous opioids due, at least in part, to decreased opioid receptors in the preBötC. Our research suggests central changes to respiratory networks responses to exogenous opioids in neonates after maternal opioids likely contribute to variability seen in breathing responses to exogenous opioids in infants with NAS during the first week of life (Jansson and Velez, 2012). In summary, our research identifies the direct effects of maternal opioids on central neonatal respiratory networks, increasing our understanding the breathing deficits in infants exposed to maternal opioids.

## Data Availability

The raw data supporting the conclusion of this article will be made available by the authors, without undue reservation.
